# Instrumented Mouthguards in Men’s Rugby League: Quantifying the Incidence and Probability of Head Acceleration Events at a Group and Individual Level

**DOI:** 10.1007/s40279-025-02253-y

**Published:** 2025-06-06

**Authors:** James Tooby, Cameron Owen, Thomas Sawczuk, Gregory Roe, Kevin Till, Gemma Phillips, Dane Vishnubala, Ryan White, Steve Rowson, Ross Tucker, Gregory Tierney, Ben Jones

**Affiliations:** 1 Carnegie Applied Rugby Research (CARR) Centre, Carnegie School of Sport, Leeds Beckett University, Leeds, UK; 2 Rugby Football League, Etihad Campus, Manchester, UK; 3 Leeds Rhinos Rugby League Club, Leeds, UK; 4 Hull Kingston Rovers Rugby League Club, Hull, UK; 5 Faculty of Biological Sciences School of Biomedical Sciences, University of Leeds, Leeds, UK; 6 School of Public Health, Imperial College London, London, UK; 7 Biomedical Engineering and Mechanics, Virginia Tech, Blacksburg, VA 24060 USA; 8 World Rugby, 8-10 Pembroke St., Dublin, Ireland; 9 Institute of Sport and Exercise Medicine, Department of Sport Science, University of Stellenbosch, Stellenbosch, South Africa; 10 Nanotechnology and Integrated Bioengineering Centre (NIBEC), School of Engineering, Ulster University, Belfast, UK; 11 Division of Physiological Sciences, Department of Human Biology, Faculty of Health Sciences, University of Cape Town, Cape Town, South Africa; 12 School of Behavioural and Health Sciences Faculty of Health Sciences, Australian Catholic University, Brisbane, QLD Australia; 13 Premiership Rugby, London, UK

## Abstract

**Background:**

There is growing concern that exposure to head acceleration events (HAEs) may be associated with long-term neurological effects.

**Objectives:**

To quantify the incidence and probability of HAEs during men’s professional rugby league match-play on a group and individual basis using instrumented mouthguards (iMGs).

**Methods:**

A total of 91 men’s professional rugby league players participating in the 2023 Super League season wore iMGs, resulting in the collection of 775 player matches (mean 8.3 matches per player). Incidence of HAEs (rate of HAEs per median playing time) was calculated via generalised linear mixed models. Probability of HAEs (likelihood of experiencing an HAE during a tackle-event) was calculated using an ordinal mixed effects regression model.

**Results:**

The mean incidence of HAEs exceeding 25 *g* per median playing time ranged from 0.86–1.88 for back positions and 1.83–2.02 for forward positions. The probability of exceeding 25 *g* during a tackle event was higher for ball-carriers (6.29%, 95% confidence intervals [CI] 5.27–7.58) than tacklers (4.26%, 95% CI 3.48–5.26). Several players exhibited considerably higher incidence and probability than others, e.g. one player averaged 5.02 HAEs exceeding 25 *g *per median playing time and another had a probability of 20.00% of exceeding 25 *g* during a tackle event as a ball-carrier and 34.78% as a tackler.

**Conclusions:**

This study quantifies the incidence and probability of HAEs in men’s rugby league match-play, advancing our understanding of HAE exposure in men’s rugby league. These findings support the development of individualised HAE mitigation strategies targeted at individuals with elevated HAE exposures.

**Supplementary Information:**

The online version contains supplementary material available at 10.1007/s40279-025-02253-y.

## Key Points

**Table:** 

The incidence of head acceleration events (HAEs) exceeding 25 g per median playing time ranged from 0.86 to 1.88 for backs and from 1.83 to 2.02 for forwards.
The probability of recording an HAE exceeding 25 g during tackle events was greater for ball-carriers (6.29%, 95% CI 5.27–7.58) than tacklers (4.26%, 95% CI 3.48–5.26).
Variability between individuals was demonstrated in both HAE incidence and probability, with several players exhibiting elevated values with respect to the group averages.

## Introduction

Head acceleration events (HAEs) are acceleration responses of the head caused by short-duration external collision forces [1]. Concerns exist regarding the potential association between exposure to HAEs and the development of chronic traumatic encephalopathy [2, 3] (CTE) and other neurological disorders [4, 5]. Sensor-based HAE incidence has been used to estimate the cumulative HAE exposure experienced by former American football players [2, 6]. Notably, in both studies, estimated HAE exposure over an entire career outperformed duration of play and the number of reported concussions in predicting both CTE pathology [2] and poorer performance in neuropsychological assessments [6]. As such, the use of wearable head acceleration sensors in sport can be important for understanding the implications of HAE exposure.

Rugby league is a contact sport involving a high number of tackles [7] and a relatively high concussion rate [8] compared with other sports [9], which raises concerns about HAE exposure. These concerns have prompted the implementation of instrumented mouthguards (iMGs) in rugby league [10]. Owing to their tight coupling with the skull [11] and embedded accelerometers and gyroscopes, iMGs enable the estimation of both linear and angular head kinematics [12], making them an effective means for approximating HAEs [13]. A study using iMGs in a single rugby league team reported the median magnitude of HAEs during matches [14]. The study also found that 98% of HAEs were recorded by tacklers and ball-carriers during the tackle event. In addition to its small sample from a single team, this study was limited for understanding HAE exposure in rugby league, because it did not report the incidence or probability of HAEs.

The incidence of HAEs (rate at which HAEs are recorded for a given time denominator) and the probability of HAEs from tackle-events (likelihood of an HAE being recorded during a tackle for either the ball-carrier or tackler) are important variables to report for understanding HAE exposure [12]. Reporting HAE incidence can allow for the HAE exposure of a sport or individual to be estimated on the basis of exposure time [2, 6], while HAE probability allows for sporting actions associated with the greatest risk of HAEs to be identified [15, 16]. These values have been reported previously in professional rugby union [17, 18]; however, they have yet to be quantified in professional rugby league, which is important given the technical [19] and physical [20] differences between the two rugby codes.

Quantifying the incidence and probability of HAEs in rugby league can have important implications for the practical application of iMGs. Currently, there is a lack of clinical data available to inform the interpretation of iMG outputs. For example, specific dose limits and HAE magnitude thresholds based on clinical data remain elusive [21, 22]. Under the assumption of a dose–response relationship, players with greater HAE exposures may be at an increased risk of experiencing potential negative health outcomes associated with HAE exposure [2, 6, 23]. Therefore, mean values may serve as normative data with which to assess a player’s HAE exposure relative to the population, allowing players with the highest HAE exposures to be identified and potentially managed. From this perspective, it is also important to quantify both incidence and probability of HAEs on an individual basis to explore variability within players.

The aim of this study was to quantify the incidence of HAEs in men’s professional rugby league match-play. Owing to positional differences in the number of tackles made by players per match [17], the aim was to quantify HAE incidence for each position group separately. A second aim was to quantify the probability of HAEs occurring during a tackle event for both the tackler and the ball-carrier, given that 98% of HAEs occur during the tackle event in rugby league [24]. In addition to quantifying average HAE incidence and probability, the study aimed to quantify values on an individual basis to describe variability within the sample.

## Methods

### Study Design and Participants

A prospective observational cohort study was conducted with players from all men’s teams during the 2023 Super League season as part of the Rugby Football League Tackle and Contact Kinematics, Loads and Exposure (TaCKLE) project [10], which involved all players being offered iMGs (Prevent Biometrics, Minneapolis, MN, USA) as part of a league-wide initiative. Player participation in the project was voluntary, resulting in a total of 91 participants across 12 teams and 775 player matches (Table 1). Ethics approval was received from the Leeds Beckett University Ethics Committee (ref. no. 108638).

**Table 1:** Playing positions and the number of players and player matches with instrumented mouthguard (iMG) data

	*n* Players	*n* Player matches (mean ± per player)	Median playing time (interquartile range)
*Backs*			
Fullback	5	51 (8.5 ± 8.4)	80.0 (80.0–80.0)
Wing	13	129 (8.1 ± 6.3)	80.0 (80.0–80.0)
Centre	18	136 (6.5 ± 6.0)	80.0 (80.0–80.0)
Half	7	69 (6.3 ± 6.3)	80.0 (80.0–80.0)
*Forwards*			
Prop	17	124 (7.3 ± 5.2)	41.00 (32.0–49.0)
Hooker	10	82 (8.2 ± 6.3)	54.0 (33.5–72.5)
Back row	16	126 (6.0 ± 5.0)	80.0 (61.0–80.0)
Loose forward	5	38 (3.2 ± 2.7)	52.0 (27.0–74.0)
Total	91	775 (8.3 ± 6.0)	–

The calculation of median playing time is shown in Supplementary Material Fig. 1

### Instrumented Mouthguards

Digital dental scans were conducted for each player to manufacture Custom V.1.4 Prevent Biometrics iMGs. Accelerometers and gyroscopes fitted to the iMGs sampled at 3200 Hz with measurement ranges of ± 200* g* and ± 35 rad/s, respectively. Linear and angular kinematics were written to fixed memory whenever linear acceleration at the accelerometer exceeded 8 *g *along any axis (i.e. iMGs were set with an 8 *g* per-axis trigger threshold). Each HAE was approximated with 10 ms of pre-trigger data and 40 ms of post-trigger data. Henceforth, the term HAE is used interchangeably to refer to the 50 ms of kinematics recorded by the iMG (i.e. the sensor acceleration event), which is considered a proxy measure of HAEs [12], and the in vivo event described in the Consensus Head Acceleration Measurement Practices (CHAMP) consensus definition [1].

Various post-processings of HAEs were performed by Prevent Biometrics. An in-house algorithm classified events as true positives and false positives. This algorithm has performed well in previous validations within rugby codes with positive predictive values of 0.94 [13] and 0.99 [17]; therefore, only true positives classified by this algorithm were used in the study (*n* = 17,077). Prevent Biometrics processing also included filtering the signal using a four-pole, zero-phase, low-pass Butterworth filter with a cut-off frequency (− 6 dB) of 200 Hz. Subsequently, an in-house Prevent Biometrics algorithm classified the level of noise/artefact that remained in each HAE as minimal (class 0, *n* = 15,797), moderate (class 1, *n* = 945) or severe (class 2, *n* = 335). Events classified as moderate or severe were then filtered again using 100 and 50 Hz cut-off frequencies, respectively. Linear kinematics were transformed to the estimated head centre-of-gravity (CoG) using the relative acceleration equation (Eq. 1). In addition to kinematic data, iMGs provided start and end timestamps of on-the-teeth periods via infrared proximity sensors. These data inform various in-house Prevent Biometrics algorithms, including the classification of true and false positives, and were also used by the research team to identify when the iMG was being worn throughout matches:

1 $$\overrightarrow {{a_{{\text{h}}} }} = \overrightarrow {{a_{{\text{m}}} }} + \vec{\alpha } \times r_{{{\text{mh}}}} + \vec{\omega } \times \left( {\vec{\omega } \times r_{{{\text{mh}}}} } \right)$$

In the relative acceleration equation, $\overrightarrow{{a}_{\text{h}}}$ is the linear acceleration at the head CoG with respect to time, $\overrightarrow{{a}_{\text{m}}}$ is the linear acceleration at the iMG sensor location with respect to time, ${\vec{\alpha }}$ is the angular acceleration with respect to time, ${r}_{\text{mh}}$ is the position vector from iMG sensor location to the head CoG and $\overrightarrow{\omega }$ is angular velocity with respect to time. The transformation vector (${r}_{\text{mh}}$) was [− 0.082, 0.009, − 0.065] in metres, as provided by Prevent Biometrics.

Following Prevent Biometrics’ post-processing, peak linear acceleration (PLA) and peak angular acceleration (PAA) were extracted from the resultant curves of each HAE by the research team. Additionally, rotational velocity change index [25] (RVCI, Eq. 2) was included owing to its high correlation with brain strain metrics [26]. Weighting factors (*x* = 1.00, *y* = 1.00 and *z* = 1.17) and the duration constraint (*t* = 10 ms) for RVCI were selected to maximise correlation with maximum principal strain on the basis of previous research [25]. RVCI values are reported in rad/s, consistent with the units used in the calculation:

2 $$\text{RVCI}= {\left.\sqrt{{R}_{x}{\left({\int }_{t1}^{t2}{a}_{x}\text{d}t\right)}^{2}+{R}_{y}{\left({\int }_{t1}^{t2}{a}_{y}\text{d}t\right)}^{2}+{R}_{z}{\left({\int }_{t1}^{t2}{a}_{z}\text{d}t\right)}^{2}}\right|}_{\text{max}}.$$

In the RVCI equation [25], ${a}_{x}$, ${a}_{y}$, ${a}_{z}$ are *x*, *y* and *z* components of angular acceleration, respectively; ${R}_{x}$, ${R}_{y}$ and ${R}_{z}$, are weighting factors about their respective axis and $t1$ and $t2$ are the initial and final integral times in which RVCI is calculated over. The values for $t1$ and $t2$ are selected to maximise the RVCI value but must be less than the duration constraint, which was 10 ms.

The relationship between HAE magnitude and clinical effects is unclear [21, 22]; therefore, results are provided for each metric across a range of thresholds to account for various potential thresholds of clinical significance [17, 27]. Nominally, PLA thresholds are presented at 15 *g* intervals from 10 *g* [28] to 70 *g*, while approximately proportional PAA and RVCI thresholds were selected on the basis of linear regression with PLA (Supplementary Material Fig. 2). The manuscript focuses on a 25 *g *threshold due to it being the lowest threshold value that is unlikely to be affected by the potential linear acceleration trigger bias [29].

### Video Analysis

**Fig. 1:**
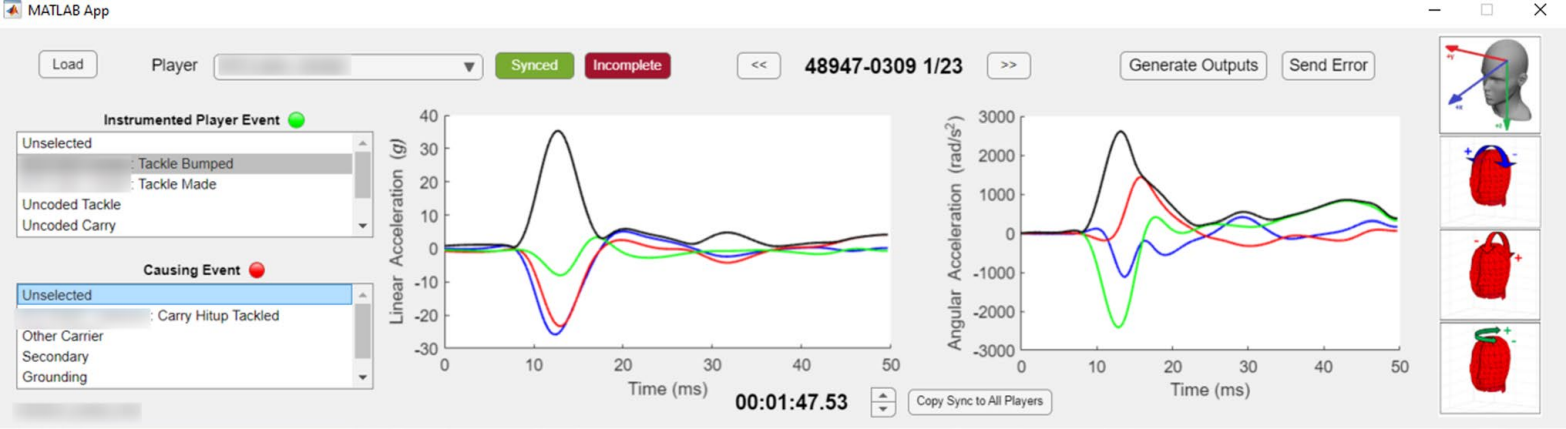
A bespoke MATLAB graphical user interface for synchronising, verifying and linking head acceleration events to Opta-coded events

Coded events from all Super League matches were obtained from Opta data provided by StatsPerform (Chicago, IL, USA). These data included events for each player tackler involvement (i.e. a player’s involvement within a tackle event as either a ball-carrier or tackler). Each coded event contained a timestamp and a player identifier. Further video analysis was conducted to identify the tackle sequence for each player tackle involvement as a tackler. Tackle sequence describes the number and order in which tacklers join a tackle event [30]: *one-on-one* tackle sequences describe a single player making initial contact with the ball-carrier; *simultaneous* tackle sequences describe two players making initial contact with the ball-carrier simultaneously; and *sequential* tackle sequences describe a player tackle involvement where a defender joins a tackle after at least one other defender has already made contact with the ball-carrier [30]. Tackler player involvements with a sequential tackle sequence were removed from the analysis as they were deemed to be tackle assists and were often included within Opta data despite there being minimal contact with the ball-carrier. This resulted in the removal of 34.2% (*n* = 2793) of tackles from the dataset. Each HAE was synchronised manually to video footage using a custom-built MATLAB graphical user interface (GUI) to adjust how HAEs from each iMG were aligned to video (Fig. 1). This process is described in a previous study as manual video-analysis based synchronisation [31]. Subsequently, video analysis was conducted using frame-by-frame playback alongside the six-degree-of-freedom kinematics visualised by the GUI. Analysts video-verified each HAE and linked it to the coded event from the Opta dataset using unique identifiers.

### Statistical Analysis

#### Incidence of Head Acceleration Events

As per recent studies [17, 18, 32, 33], only data from player matches where over 90% of player tackle involvements occurred during periods when the proximity sensor indicated the iMG was being worn were included in the dataset (Table 1). This was to reduce the likelihood of false negatives caused by poor adherence of iMG wearing or the battery of the iMG dying. A generalised linear mixed model (R package glmmTMB [34]) was used to estimate HAE incidence for forwards and backs. These models used the Poisson distribution with a logarithmic link function and used HAE count as the outcome variable, the logarithm of playing time and position as fixed effects and player identifier and fixture identifier as random effects. Separate models were used for each magnitude threshold. Incidence values were estimated from the model for each position group using two denominators: per full-match equivalent (i.e. 80 min playing time for each individual player) and per median playing time (i.e. the median playing time for each position group, calculated using player matches from the 91 players across the entire 2023 Super League season [*n* = 1631 player matches] and presented in Table 1). Individual players’ mean incidence values were estimated by adjusting the model means using each player’s individual random intercept. Each player’s median playing time across the iMG-recorded player matches was used to estimate mean incidence per median playing time.

#### Probability of Head Acceleration Events During Tackle-Events

Only player tackle involvements which occurred within the timestamps of an on-the-teeth proximity sensor period were used in the probability models (*n* = 5375 as a tackler and *n* = 4772 as a ball-carrier). The maximum magnitude of head acceleration for each Opta-coded player tackle involvement was calculated from the HAEs linked to each coded event during video analysis (Fig. 1). The maximum value for each metric type (PLA, PAA and RVCI) was selected from the HAEs linked to the player tackle involvement, and if no HAEs were linked, then the maximum magnitude value was labelled as ‘no recorded HAEs’. Probability of HAEs was estimated using an ordinal mixed effects regression model (R package ordinal [35] and clmm function). In total, three separate models were used to calculate HAE probabilities for PLA, PAA and RVCI thresholds. These models included player role (i.e. tackler or ball-carrier) and maximum magnitude as fixed effects. ‘Player ID’ nested within ‘fixture ID’ and ‘tackle event ID’ (i.e. events occurring during the same tackle event) were included as random effects. Exceedance probabilities were calculated from the model results using bootstrapping, whereby tackle involvements labelled with ‘no recorded HAEs’ were assumed not to have exceeded the trigger threshold. These probabilities estimate the likelihood of a player tackle involvement resulting in a HAE exceeding a given magnitude.

Individual exceedance probability values were calculated outside the ordinal mixed effects regression models by dividing the number of tackle involvements that exceeded a given magnitude threshold by the total number of measured tackle involvements. This was done for each player and for tackler and ball-carrier tackle involvements separately. Individual values were only calculated for individual players with greater than 15 involvements both as a tackler and ball-carrier (*n* = 62).

Across all generalised linear mixed models and ordinal mixed effects regression models, residual diagnostics of all models were assessed using the performance package in R [36]. All results from the models are reported as mean values with 95% confidence intervals (CI). Comparisons were deemed significantly different if confidence limits did not overlap [37].

## Results

### Incidence of Head Acceleration Events

**Fig. 2:**
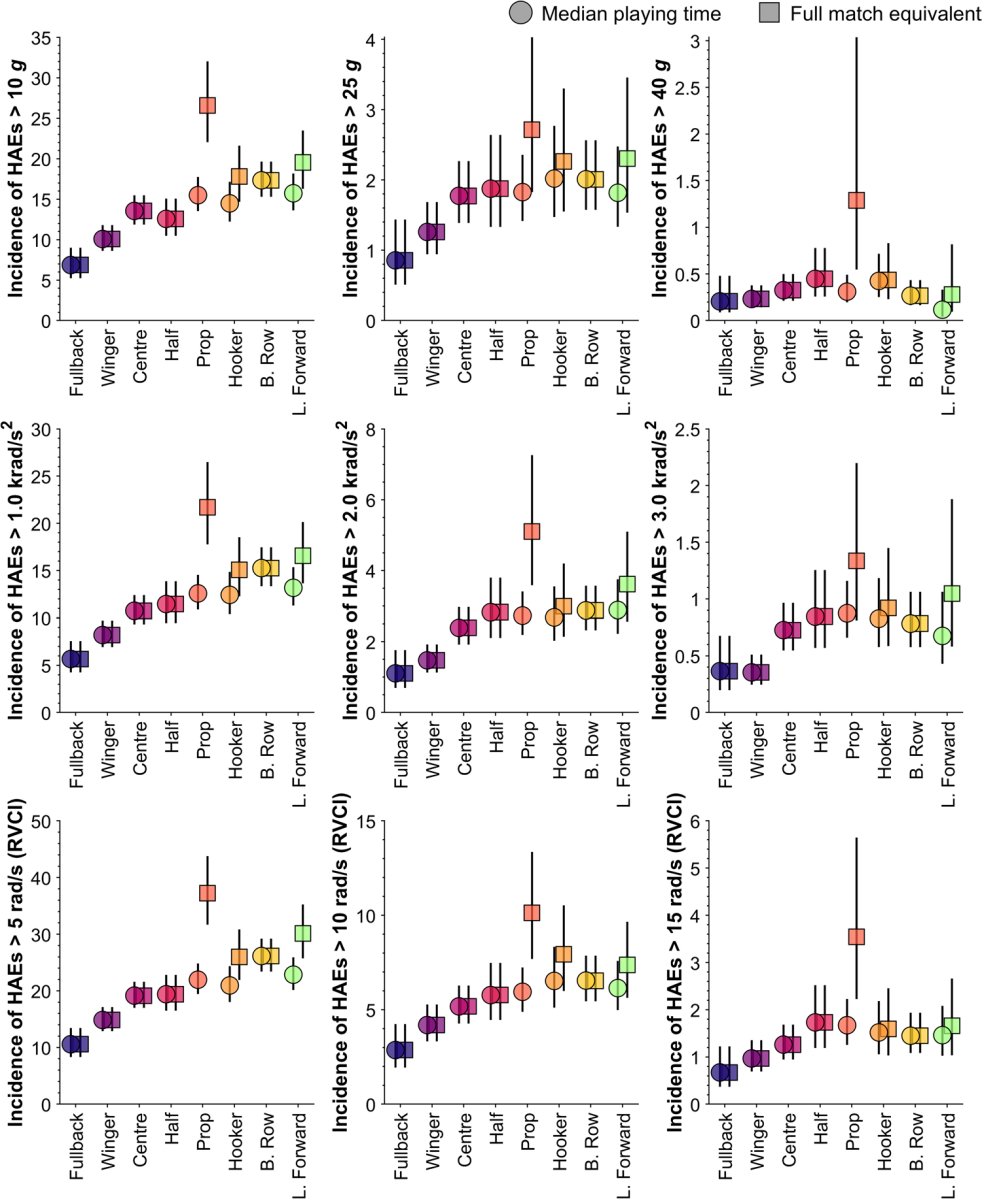
The mean incidence of HAEs for each position using two denominators: per full-match equivalent (i.e. per 80 min playing time) and per median playing time (i.e. per median playing time for each position). Lines show 95% CI. *HAEs* head acceleration events, *PLA* peak linear acceleration, *PAA* peak angular acceleration, *RVCI* rotational velocity change index

Figure 2 shows the mean incidence of HAEs exceeding a range of PLA, PAA and RVCI thresholds for each position group for two denominators: per full-match equivalent (i.e. per 80 min playing time) and per median playing time (i.e. per median playing time for each position). These values are tabulated in Supplementary Material Table 1. On average, the mean incidence of HAEs exceeding 25 *g* per median playing time ranged from 0.86 to 1.88 for back positions and 1.83 to 2.02 for forward positions. The incidence of higher magnitude HAEs was very low on average on a player-match basis. For example, the mean incidence of HAEs exceeding the highest thresholds (i.e. exceeding 70 *g*, 5000 rad/s^2^ or an RVCI of 20 rad/s) was 0.00 to 0.16 per median playing time in forward positions and 0.00 to 0.17 for back positions (Supplementary Material Table 1). In 83.97% (*n* = 634) of player matches, no HAEs exceeded those highest thresholds.

The median playing time for fullback, winger, centre, half and back row positions was 80 min (Supplementary Material Fig. 3); therefore, the HAE incidence for these positions was unaffected by the different denominators (i.e. per full-match equivalent and per median playing time). Prop, hooker and loose forwards positions had median playing times of 41, 54 and 52 min, respectively, and thus these positions had higher mean HAE incidence per full-match equivalent but lower incidences per median playing time. For example, props had a mean incidence of 21.70 (95% CI 17.77–26.49) HAEs exceeding 1000 rad/s^2^ per full-match equivalent; however, because props only played for an average of 41 min per match, the mean incidence per median playing time was 12.58 (95% CI 10.88–14.54; Fig. 2).

### Probability of Head Acceleration Events

**Fig. 3:**
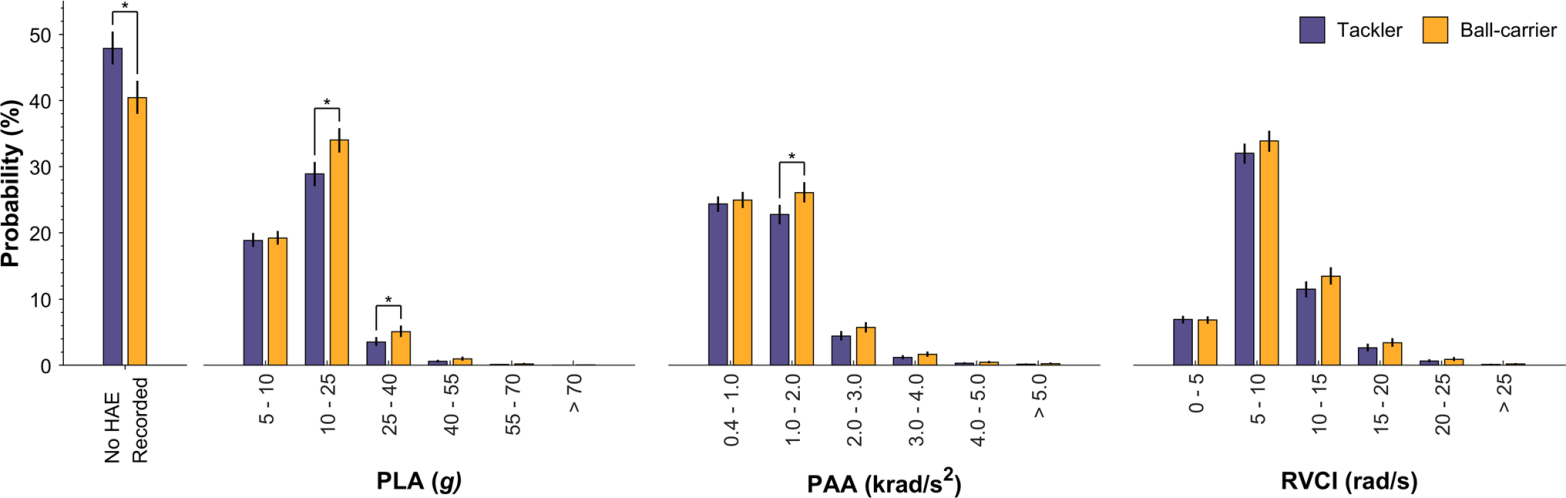
The probability of the maximum magnitude HAE occurring between a range of ordinal thresholds. Vertical lines indicate 95% confidence intervals. Asterisks indicate comparisons between tackler and ball-carrier probability with non-overlapping confidence intervals. *PLA* peak linear acceleration, *PAA* peak angular acceleration, *RVCI* rotational velocity change index

The probabilities of player tackle involvements exceeding a range of PLA, PAA and RVCI thresholds are presented in Table 2. The probability of exceeding 25 *g* was greater as a ball-carrier (6.29%, 95% CI 5.27–7.58) than as a tackler (4.26%, 95% CI 3.48–5.26). Figure 3 shows the probability of the maximum HAE magnitude from a player tackle involvement for a range of ordinal thresholds for ball-carrier and tackler events separately. Tackler events were more likely to result in no recorded HAEs than ball-carrier events, while ball-carrier events were significantly more likely to result in a maximum HAE magnitude between 10 and 25 g, 25 and 40 *g* and 1000 and 2000 rad/s^2^. On average, the probability of a higher-magnitude HAE, for example, exceeding 5000 rad/s^2^, was very low for both the ball-carrier and tackler, at 0.25% (95% CI 0.15–0.42) and 0.16% (95% CI 0.09–0.27), respectively.

**Table 2:** The probability (95% CI) of ball-carriers and tacklers experiencing an HAE exceeding a given threshold during a tackle event

Metric	Threshold	Ball-carrier	Tackler
PLA	> 10 *g*	40.31% (37.93–42.81)	33.19% (30.85–35.59)
	> 25 *g*	6.29% (5.27–7.58)	4.26% (3.48–5.26)
	> 40 g	1.23% (0.89–1.69)	0.74% (0.51–1.05)
	> 55 *g*	0.27% (0.16–0.45)	0.15% (0.08–0.26)
	> 70 *g*	0.05% (0.02–0.13)	0.03% (0.01–0.07)
PAA	> 1000 rad/s^2^	34.14% (32.05–36.44)	28.78% (26.64–31.06)
	> 2000 rad/s^2^	8.07% (6.83–9.47)	6.04% (5.00–7.22)
	> 3000 rad/s^2^	2.36% (1.80–3.03)	1.62% (1.19–2.17)
	> 4000 rad/s^2^	0.70% (0.48–1.03)	0.46% (0.29–0.69)
	> 5000 rad/s^2^	0.25% (0.15–0.42)	0.16% (0.09–0.27)
RVCI	> 5 rad/s	51.82% (49.15–54.29)	46.84% (44.43–49.42)
	> 10 rad/s	17.88% (15.94–20.03)	14.91% (13.16–16.79)
	> 15 rad/s	4.46% (3.59–5.46)	3.42% (2.69–4.24)
	> 20 rad/s	1.08% (0.76–1.50)	0.78% (0.54–1.11)
	> 25 rad/s	0.20% (0.11–0.34)	0.13% (0.07–0.24)

*PLA* peak linear acceleration, *PAA* peak angular acceleration, *RVCI* rotational velocity change index

### Individual Head Acceleration Event Incidence and Probability

**Fig. 4:**
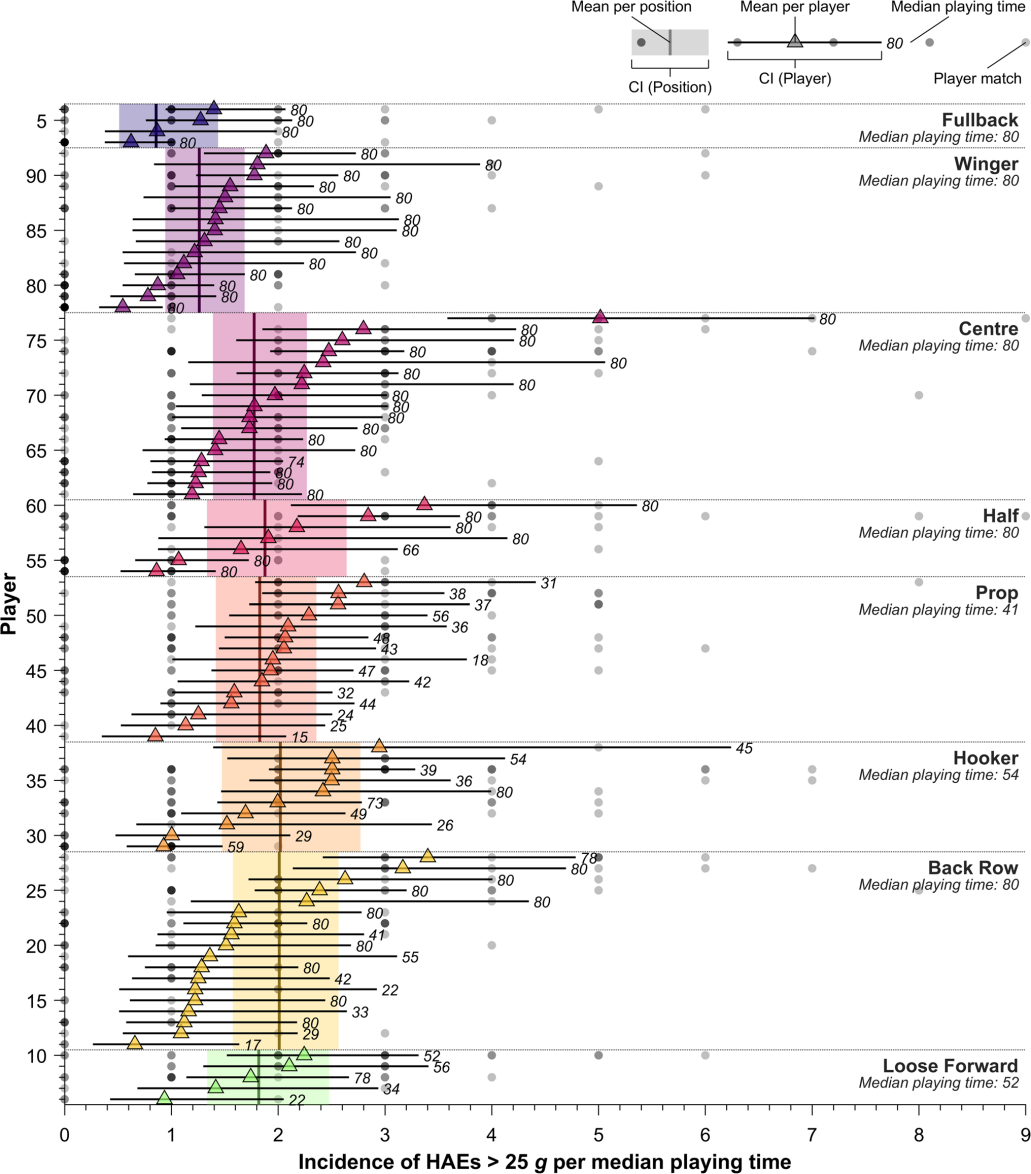
Mean incidence of HAEs exceeding 25 *g* per median playing time for each player and each position and individual counts of HAEs per player match. The numbers at the end of each confidence interval show the median playing time for each player. The circles along each player’s row are individual HAE counts for their matches; multiple matches with the same HAE count are indicated by darker circles. This figure is shown for other thresholds in Supplementary Material Figs. 3–10. *HAEs* head acceleration events

**Fig. 5:**
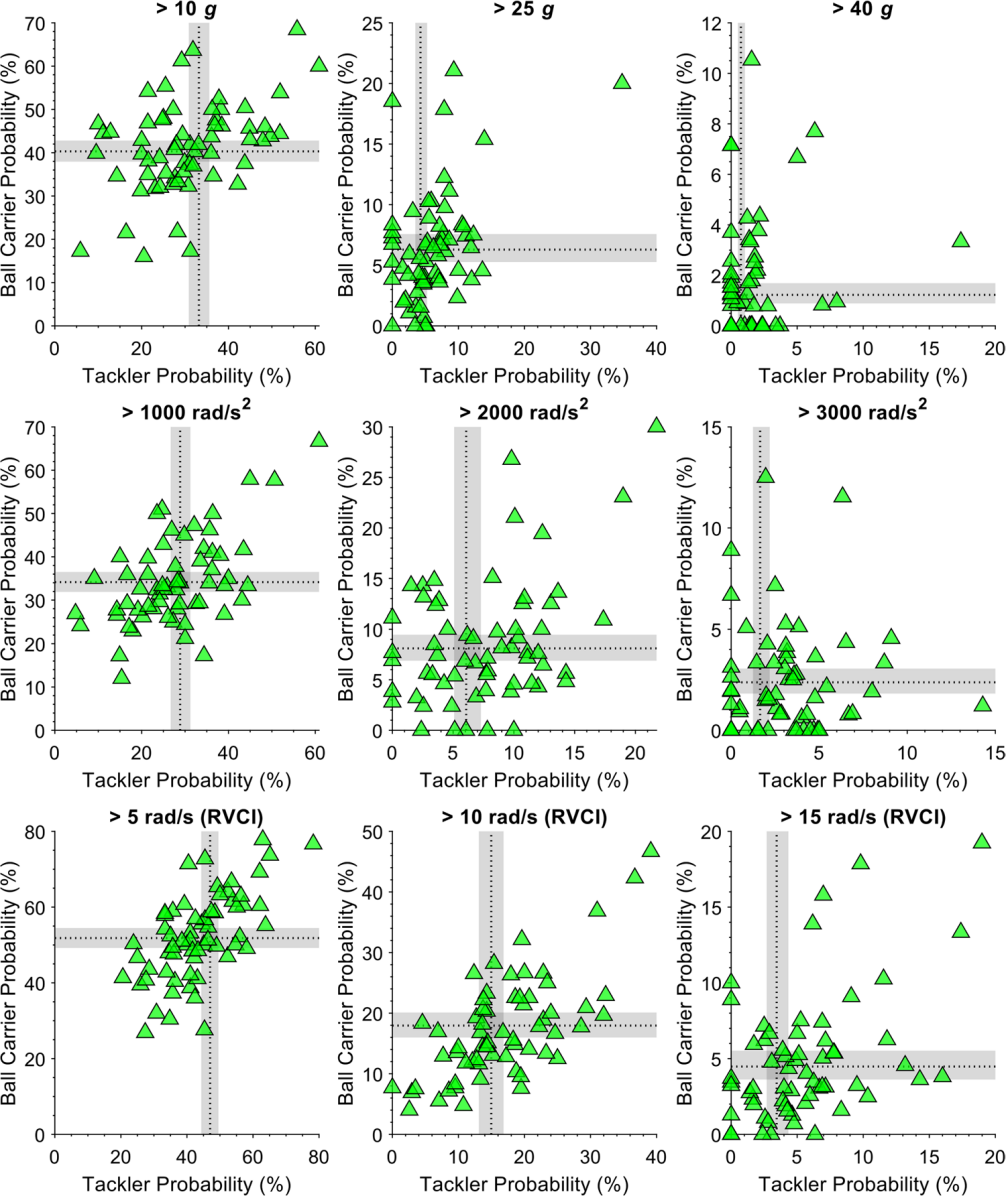
Each player’s probability of an HAE exceeding a range of PLA, PAA and RVCI thresholds as a ball-carrier (*y* axis) and as a tackler (*x* axis). Thresholds are indicated in the individual panel (or graph) titles. Only players with more than 15 involvements as both a tackler and ball-carrier were included (*n* = 62). Shaded areas are the modelled probabilities estimated by the ordinal mixed effects regression model with confidence intervals. *HAE* head acceleration event, *PLA* peak linear acceleration, *PAA* peak angular acceleration, *RVCI* rotational velocity change index

The variability between individuals in HAE incidence and probability are clearly demonstrated in Figs. 4 and 5. Figure 4 shows the mean HAE incidence values per median playing time for individual players, with each player’s denominator based on their median playing time across the entire season and displayed as text in the figure. The mean incidence of one individual (player 72, centre) was 5.02 (95% CI 3.58–7.00) HAEs exceeding 25 g per median playing time, which was almost threefold higher than the mean HAE incidence of another centre (1.77, 95% CI 1.39–2.27). Another player (player 48, prop) had a mean incidence of 2.81 (95% CI 1.78–4.41) HAEs exceeding 25 *g* per median playing time, despite having a median playing time of just 31 min per match. Figure 5 shows each player’s individual probability of recording an HAE exceeding a given threshold as a tackler (*x* axis) against their probability as a ball-carrier (*y* axis). In total, five players had a probability of exceeding 25 *g* as a ball-carrier above 15.00%, despite the overall modelled probability being 6.29% (95% CI 5.27–7.58%). One player had a probability of 20.00% of exceeding 25 *g* during a tackle event as a ball-carrier and 34.78% as a tackler, which was more than three and eight times greater than the modelled probability as a tackler and ball-carrier, respectively.

## Discussion

The aim of this study was to quantify the incidence and probability of HAEs during men’s rugby league match-play per position group and on an individual basis. Incidence of HAEs is provided across a range of thresholds for each position (e.g. mean incidence of HAEs exceeding 25 g per median playing time ranged from 0.86 to 1.88 for back positions and 1.83 to 2.02 for forward positions). The probability of an HAE occurring was greater for ball-carriers than for tacklers but was low for both groups of at higher thresholds (e.g. lower than 0.30% probability of exceeding 5000 rad/s^2^). Finally, mean HAE incidence and probability values were quantified and visualised on an individual basis, clearly demonstrating variability between individuals, with some players exhibiting elevated values compared with group averages. These findings advance our understanding of HAE exposure in men’s rugby league, providing reference data from which to inform the interpretation of HAEs in the future. These data may also support the development of individualised HAE mitigation strategies in men’s rugby league, revealing that targeting interventions at those individuals with the highest HAE exposures may be necessary.

This study found that the incidence of HAEs was greater in forwards than in backs and that ball-carriers were more likely to experience HAEs in a tackle event than carriers. Previous studies in rugby union also identified that HAEs occurred at a higher rate in forwards than backs [17]; however, the probability of recording an HAE was greater in tacklers than carriers [17, 32]. Interestingly, on average, both HAE incidence and probability appear lower in this cohort of men’s professional rugby league players compared with previous cohorts of men’s professional rugby union players [17]. The incidence of HAEs exceeding 25 *g *per full match equivalent in men’s rugby union was greater than documented here (i.e. in rugby union versus the present study; forwards: 5.42 versus 2.16 and backs: 3.92 versus 1.51) [17]. Furthermore, the probability of exceeding 25 *g* during a tackle event was 18.90% and 13.90% for tacklers and ball-carriers, respectively, in a similar men’s rugby union cohort [32], compared with 4.26% and 6.29% in the present study, with both studies using the same statistical approach, iMG system and video analysis data. Lower probabilities may be observed in rugby league compared with rugby union owing to numerous differences between the rugby codes. Speculatively, these may include differences in the physical characteristics of league and union players or the differences in the laws between codes (e.g. the 10-m retreat rule in rugby league) which may influence player behaviour and technique. As sports work to mitigate HAE exposure within their unique constraints, cross-sport collaboration establishing differences in HAE exposure and identifying unique or shared causal mechanisms to explain the potential differences may be useful to inform potential mitigation strategies.

This study demonstrated that some players exhibited mean HAE incidence values that were almost three times the population average (Fig. 4). If this trend were replicated across multiple matches and seasons, it would lead to some players experiencing far greater HAE exposures than others. Future research is needed to confirm whether some players are likely to experience a greater HAE exposure than others across a more extended time period. Under the assumption of a dose–response relationship, individuals with the highest HAE exposure may be at the greatest risk of developing the potential long-term effects from HAE exposure [2, 6, 23] but may also be exhibiting behaviours that increase acute risks of head injury (e.g. concussion). The clinical monitoring of players with elevated HAE exposures may be beneficial to detect any potential short-term effects of HAE exposure. Mitigation strategies focused on individuals with the highest HAE exposures may be necessary for preventing excessive HAE exposures and their potential immediate and chronic effects. These mitigation strategies may focus on reducing the players’ probability of recording HAEs during contact events, for example, promoting safer tackle technique through coaching. Similar interventions have been conducted in youth American football with successful results [38, 39]. Alternatively, HAE exposure may be prevented entirely by reducing exposure time from a player management perspective. The Super League has implemented playing time limits of 30 full-match equivalents per season (i.e. 2400 min) for backs and 25 full match equivalents per season (i.e. 2000 min) for forwards on the basis of preliminary data from this study [40]. Given that iMGs are now mandated in this competition, individual HAE exposure may, in the future, inform the individual playing time limits based on iMG data.

The cause of the higher HAE incidence in some players is unknown; however, it is likely influenced by both the per-match exposure to collisions and the variability between individuals in their probability of experiencing HAEs during tackle events, which was highly variable between players (Fig. 5). This suggests that there are individual factors that influence the probability of an HAE and that may contribute either separately or additively to overall HAE incidence. Speculatively, these may include individual characteristics such as player age, playing standard or experience; physical factors such as neck strength [41–43], body size or other physical qualities and technical factors such as tackle technique and technical demands imposed by team and position. These were all beyond the scope of this study but invite future research with the intention of understanding the basis for this variability so that interventions may be targeted to reduce the overall HAE incidence. Such interventions [44] may have varying degrees of success or indeed adoption [45]. Future research elucidating the influence of individual factors can be important for informing future interventions from a range of stakeholder perspectives (e.g. strength and conditioning interventions aimed at improving physical qualities or coaching interventions [46] aimed at improving technical factors associated with a lower probability). At the simplest level, however, reducing the overall playing duration (i.e. match exposure) for individuals that accumulate the highest HAE exposure may be the most effective mitigation strategy. Despite this, it may also be the least feasible given the performance demands of professional sport. Reductions in player exposure may be supported by iMG data if these data are used to inform player management at each respective club or to inform player match limits set by sports governing bodies [40].

### Limitations

This is the first league-wide iMG study to quantify HAE incidence and probability in professional men’s rugby league; however, the clinical implications of these findings are limited by the lack of understanding as to the clinical effects of HAE magnitudes [21, 22]. Specifically, the magnitude of HAE that is clinically significant from a cumulative exposure perspective is unknown. Consequently, this study provides findings across a range of thresholds to accommodate a range of assumptions as to which magnitudes are clinically significant. Metrics quantifying the degree of brain deformation may be more effective for approximating injury risk than peak kinematics that ignore pulse duration and directionality (i.e. PLA and PAA); therefore, RVCI was included as it has previously demonstrated high correlation with brain strain [26].

Second, it is important to acknowledge that all iMG systems that utilise linear acceleration trigger mechanisms to record HAEs suffer from a potential trigger bias that causes false negatives [12, 29]. Certain impact conditions, particularly impact locations to the front and top of the head, can result in a magnitude of linear acceleration that is relatively low at the iMG sensor location compared with that at the head centre-of-gravity [29]. Simulations identified that the bias can cause HAEs up to 30 *g* to be missed when using a 10 *g* trigger threshold [29]. The iMG system in the present study used an 8 *g* trigger threshold; therefore, it is assumed that false negatives may have occurred up to approximately 25 *g*. Consequently, the discussion of findings in this study focusses on HAEs exceeding 25 *g* to minimise the risk of false negatives. However, false negatives may still occur from other mechanisms, such as the rearming period [12]. Angular thresholds of 2000 rad/s^2^ and 10 rad/s (RVCI) are proportional to 25 *g*; however, HAE incidence and probability values may still contain false negatives caused by the linear bias owing to HAEs being proportionally lower in linear acceleration than angular metrics (PAA or RVCI). Other technical limitations of iMGs may also limit the accuracy of kinematics reported by the iMGs, specifically, the use of 50 or 100 Hz filters in 8.10% (*n* = 1280) of HAEs may have led to underestimated magnitudes in this sample of HAEs [47].

Finally, this study focused only on men’s rugby league matchplay. Further research is required to quantify HAEs in training and across other rugby league levels and contexts, including women’s competitions. Furthermore, probablility values in this study are limited to the initial tacklers; therefore, the probability of HAEs from tackle assists was not quantified.

## Conclusions and Policy Implications

This study quantifies the incidence and probability of HAEs in men’s rugby league match play for the first time. Incidence is provided across a range of thresholds for each position (e.g. mean incidence of HAEs exceeding 25 g per median playing time ranged from 0.86 to 1.88 for back positions and 1.83 to 2.02 for forward positions), providing reference data from which to inform the interpretation of HAEs in the future. The probability of an HAE was greater for ball-carriers than for tacklers (6.29% versus 4.26%) but was low for both players at higher thresholds (0.16% and 0.25% for tacklers and ball-carriers, respectively). Finally, this study demonstrated variability between players in both HAE incidence and probability, with some players exhibiting elevated values with respect to the group averages. Due to a proposed dose–response relationship between HAE exposure and long-term effects, individuals with the greatest HAE exposure may be at the greatest risk of potential long-term effects. Consequently, these findings may support the need for individualised monitoring and management of HAE exposure, targeting those individuals with elevated HAE exposures.

The findings of this study support the development of individualised HAE mitigation strategies in men’s rugby league, owing to the demonstrated variability between individuals. By focusing on those with the highest HAE exposures, it may be possible to prevent excessive HAEs and their potential long-term effects. Therefore, individualised monitoring and mitigation strategies may be crucial. Future policies could include clinical monitoring for short-term effects and interventions aimed at reducing exposure through modified playing duration, improved physical conditioning or enhanced tackle techniques. Cross-sport collaboration to understand differences in HAE exposure and identifying shared causal mechanisms may further inform effective mitigation strategies.

## Supplementary Information

Below is the link to the electronic supplementary material.

Supplementary file1 (PDF 1363 KB)

## Declarations

### Patient and Public Involvement

Patients and/or the public were not involved in the design, conduct, reporting or dissemination plans of this research.

### Data Sharing

All data relevant to the study are included in the article or uploaded as supplementary information. Anonymised data should be available upon reasonable request.
