# Supplementary material for: Instrumented Mouthguards in Men’s Rugby League: Quantifying the Incidence and Probability of Head Acceleration Events at a Group and Individual Level
Source: Sports Med. 2025 Jun 6;55(11):2879–90. doi: 10.1007/s40279-025-02253-y (PMC12559052; doi:10.1007/s40279-025-02253-y)
Supplement: Supplementary file 1 — Supplementary file1 (PDF 1363 KB) [file 40279_2025_2253_MOESM1_ESM.pdf]

Instrumented Mouthguards in Men's Rugby League: Quantifying the Incidence and Probability of Head Acceleration Events at a Group and Individual Level

Supplementary Materials

Supplementary Table 1. Tabulated incidence values (with CI) for each position group across various PLA (*g*), PAA (rad/s^2), and RVCI (rad/s) thresholds.

Threshold	Centre	Prop	Hooker	Back Row	Loose Forward	Winger	Half	Fullback
Incidence per typical match								
> 10 *g*	13.54 (11.86 - 15.47)	15.49 (13.52 - 17.74)	14.48 (12.23 - 17.14)	17.33 (15.29 - 19.64)	15.72 (13.61 - 18.16)	10.07 (8.61 - 11.79)	12.56 (10.47 - 15.06)	6.86 (5.23 - 8.99)
> 25 *g*	1.77 (1.39 - 2.27)	1.83 (1.42 - 2.36)	2.02 (1.47 - 2.77)	2.01 (1.58 - 2.57)	1.82 (1.33 - 2.48)	1.26 (0.94 - 1.69)	1.88 (1.33 - 2.64)	0.86 (0.51 - 1.44)
> 40 *g*	0.33 (0.21 - 0.50)	0.31 (0.20 - 0.49)	0.42 (0.25 - 0.72)	0.27 (0.16 - 0.43)	0.12 (0.04 - 0.33)	0.23 (0.14 - 0.38)	0.45 (0.26 - 0.78)	0.21 (0.09 - 0.48)
> 55 *g*	0.11 (0.06 - 0.22)	0.11 (0.05 - 0.22)	0.07 (0.03 - 0.18)	0.05 (0.02 - 0.13)	0.04 (0.01 - 0.22)	0.06 (0.02 - 0.13)	0.12 (0.05 - 0.28)	0.10 (0.03 - 0.29)
> 70 *g*	0.04	0.02	0.01	0.00	0.00	0.00	0.01	0.05
> 1,000 rad/s^2	10.75 (9.32 - 12.41)	12.58 (10.88 - 14.54)	12.42 (10.39 - 14.86)	15.26 (13.34 - 17.46)	13.17 (11.30 - 15.36)	8.21 (6.93 - 9.72)	11.45 (9.46 - 13.87)	5.67 (4.25 - 7.56)
> 2,000 rad/s^2	2.39 (1.92 - 2.98)	2.73 (2.19 - 3.41)	2.68 (2.03 - 3.55)	2.88 (2.32 - 3.57)	2.89 (2.22 - 3.75)	1.47 (1.13 - 1.92)	2.83 (2.10 - 3.80)	1.11 (0.69 - 1.76)
> 3,000 rad/s^2	0.73 (0.55 - 0.97)	0.87 (0.66 - 1.16)	0.83 (0.58 - 1.18)	0.78 (0.58 - 1.06)	0.68 (0.43 - 1.06)	0.35 (0.25 - 0.51)	0.85 (0.57 - 1.25)	0.37 (0.20 - 0.68)
> 4,000 rad/s^2	0.24 (0.16 - 0.36)	0.30 (0.19 - 0.47)	0.35 (0.22 - 0.55)	0.19 (0.12 - 0.31)	0.20 (0.09 - 0.44)	0.11 (0.07 - 0.20)	0.19 (0.08 - 0.46)	0.14 (0.06 - 0.32)
> 5,000 rad/s^2	0.07 (0.04 - 0.15)	0.08 (0.04 - 0.17)	0.10 (0.05 - 0.23)	0.08 (0.03 - 0.16)	0.01 (0.00 - 1.25)	0.03 (0.01 - 0.09)	0.07 (0.03 - 0.20)	0.04 (0.01 - 0.17)
> 5 rad/s	19.16 (17.00 - 21.60)	21.97 (19.44 - 24.82)	20.96 (18.03 - 24.36)	26.14 (23.40 - 29.21)	22.85 (20.15 - 25.92)	14.86 (12.88 - 17.14)	19.40 (16.50 - 22.81)	10.57 (8.32 - 13.44)
> 10 rad/s	5.18 (4.27 - 6.28)	5.95 (4.89 - 7.24)	6.52 (5.11 - 8.33)	6.54 (5.45 - 7.86)	6.15 (4.98 - 7.58)	4.19 (3.33 - 5.28)	5.77 (4.46 - 7.48)	2.86 (1.94 - 4.24)
> 15 rad/s	1.26 (0.95 - 1.68)	1.67 (1.26 - 2.23)	1.52 (1.06 - 2.18)	1.45 (1.08 - 1.94)	1.46 (1.03 - 2.08)	0.97 (0.69 - 1.35)	1.73 (1.19 - 2.52)	0.67 (0.37 - 1.22)
> 20 rad/s	0.37 (0.26 - 0.53)	0.46 (0.33 - 0.64)	0.45 (0.29 - 0.69)	0.39 (0.26 - 0.57)	0.38 (0.22 - 0.67)	0.20 (0.12 - 0.31)	0.51 (0.32 - 0.80)	0.27 (0.14 - 0.54)
> 25 rad/s	0.13 (0.07 - 0.21)	0.08 (0.04 - 0.15)	0.12 (0.06 - 0.25)	0.16 (0.09 - 0.27)	0.06 (0.02 - 0.23)	0.03 (0.01 - 0.08)	0.17 (0.10 - 0.31)	0.07 (0.02 - 0.25)
Incidence per full match equivalent								
> 10 *g*	13.54 (11.86 - 15.47)	26.57 (22.04 - 32.02)	17.80 (14.66 - 21.61)	17.33 (15.29 - 19.64)	19.55 (16.27 - 23.48)	10.07 (8.61 - 11.79)	12.56 (10.47 - 15.06)	6.86 (5.23 - 8.99)
> 25 *g*	1.77 (1.39 - 2.27)	2.72 (1.83 - 4.03)	2.26 (1.55 - 3.30)	2.01 (1.58 - 2.57)	2.30 (1.54 - 3.46)	1.26 (0.94 - 1.69)	1.88 (1.33 - 2.64)	0.86 (0.51 - 1.44)
> 40 *g*	0.33 (0.21 - 0.50)	1.29 (0.55 - 3.04)	0.44 (0.23 - 0.83)	0.27 (0.16 - 0.43)	0.28 (0.09 - 0.82)	0.23 (0.14 - 0.38)	0.45 (0.26 - 0.78)	0.21 (0.09 - 0.48)
> 55 *g*	0.11 (0.06 - 0.22)	0.32 (0.09 - 1.12)	0.12 (0.04 - 0.36)	0.05 (0.02 - 0.13)	0.08 (0.01 - 0.47)	0.06 (0.02 - 0.13)	0.12 (0.05 - 0.28)	0.10 (0.03 - 0.29)
> 70 *g*	0.04	0.02	0.01	0.00	0.00	0.00	0.01	0.05
> 1,000 rad/s^2	10.75 (9.32 - 12.41)	21.70 (17.77 - 26.49)	15.08 (12.27 - 18.53)	15.26 (13.34 - 17.46)	16.58 (13.65 - 20.14)	8.21 (6.93 - 9.72)	11.45 (9.46 - 13.87)	5.67 (4.25 - 7.56)
> 2,000 rad/s^2	2.39 (1.92 - 2.98)	5.10 (3.59 - 7.26)	3.00 (2.14 - 4.20)	2.88 (2.32 - 3.57)	3.61 (2.56 - 5.10)	1.47 (1.13 - 1.92)	2.83 (2.10 - 3.80)	1.11 (0.69 - 1.76)
> 3,000 rad/s^2	0.73 (0.55 - 0.97)	1.34 (0.81 - 2.20)	0.92 (0.59 - 1.45)	0.78 (0.58 - 1.06)	1.05 (0.58 - 1.88)	0.35 (0.25 - 0.51)	0.85 (0.57 - 1.25)	0.37 (0.20 - 0.68)
> 4,000 rad/s^2	0.24 (0.16 - 0.36)	0.53 (0.24 - 1.19)	0.42 (0.24 - 0.75)	0.19 (0.12 - 0.31)	0.36 (0.15 - 0.86)	0.11 (0.07 - 0.20)	0.19 (0.08 - 0.46)	0.14 (0.06 - 0.32)
> 5,000 rad/s^2	0.07 (0.04 - 0.15)	0.17 (0.04 - 0.65)	0.19 (0.07 - 0.51)	0.08 (0.03 - 0.16)	0.17 (0.04 - 0.79)	0.03 (0.01 - 0.09)	0.07 (0.03 - 0.20)	0.04 (0.01 - 0.17)
> 5 rad/s	19.16 (17.00 - 21.60)	37.24 (31.68 - 43.78)	26.00 (21.91 - 30.84)	26.14 (23.40 - 29.21)	30.11 (25.73 - 35.24)	14.86 (12.88 - 17.14)	19.40 (16.50 - 22.81)	10.57 (8.32 - 13.44)
> 10 rad/s	5.18 (4.27 - 6.28)	10.13 (7.69 - 13.35)	7.94 (5.99 - 10.53)	6.54 (5.45 - 7.86)	7.37 (5.63 - 9.66)	4.19 (3.33 - 5.28)	5.77 (4.46 - 7.48)	2.86 (1.94 - 4.24)
> 15 rad/s	1.26 (0.95 - 1.68)	3.54 (2.23 - 5.65)	1.59 (1.03 - 2.45)	1.45 (1.08 - 1.94)	1.66 (1.03 - 2.66)	0.97 (0.69 - 1.35)	1.73 (1.19 - 2.52)	0.67 (0.37 - 1.22)
> 20 rad/s	0.37 (0.26 - 0.53)	0.80 (0.41 - 1.55)	0.43 (0.25 - 0.75)	0.39 (0.26 - 0.57)	0.46 (0.21 - 0.98)	0.20 (0.12 - 0.31)	0.51 (0.32 - 0.80)	0.27 (0.14 - 0.54)
> 25 rad/s	0.13 (0.07 - 0.21)	0.23 (0.06 - 0.97)	0.10 (0.04 - 0.26)	0.16 (0.09 - 0.27)	0.04 (0.01 - 0.25)	0.03 (0.01 - 0.08)	0.17 (0.10 - 0.31)	0.07 (0.02 - 0.25)

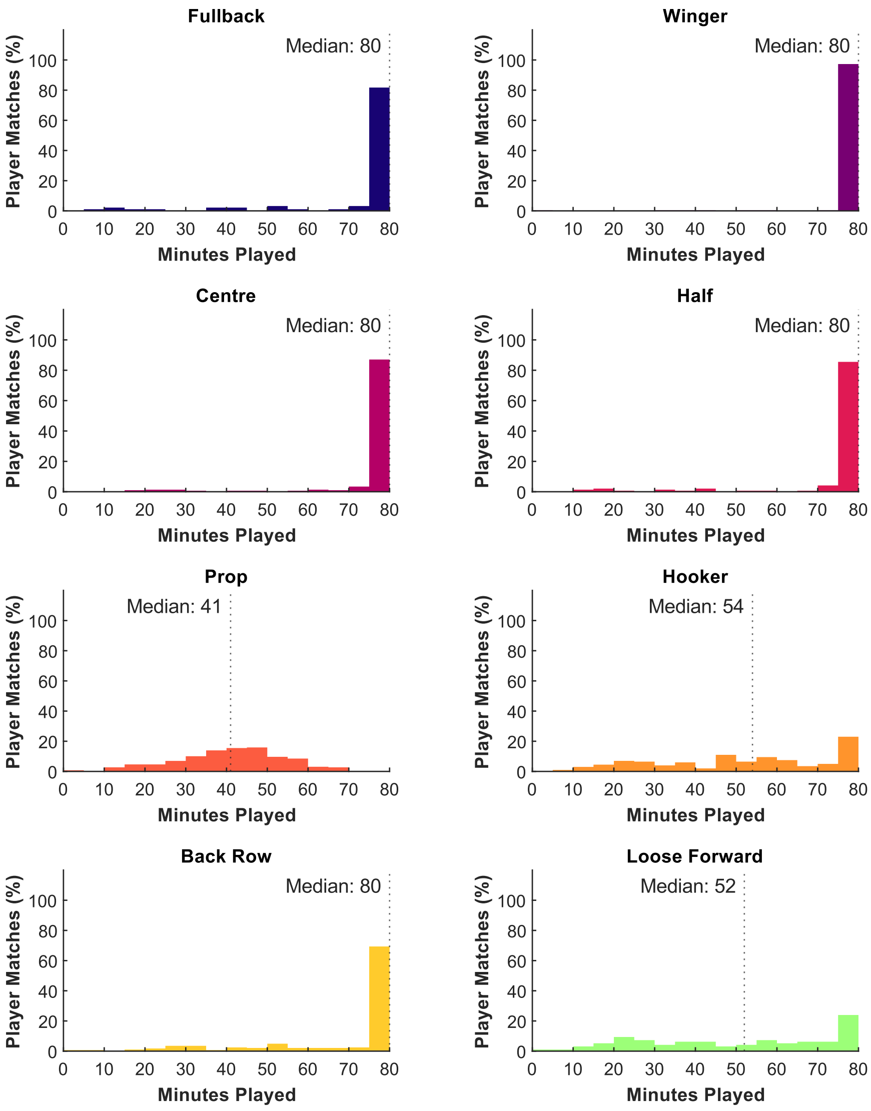

Supplementary Fig. 1 Median minutes played per player match for each position group. These data were used to calculate incidence *per typical match*.

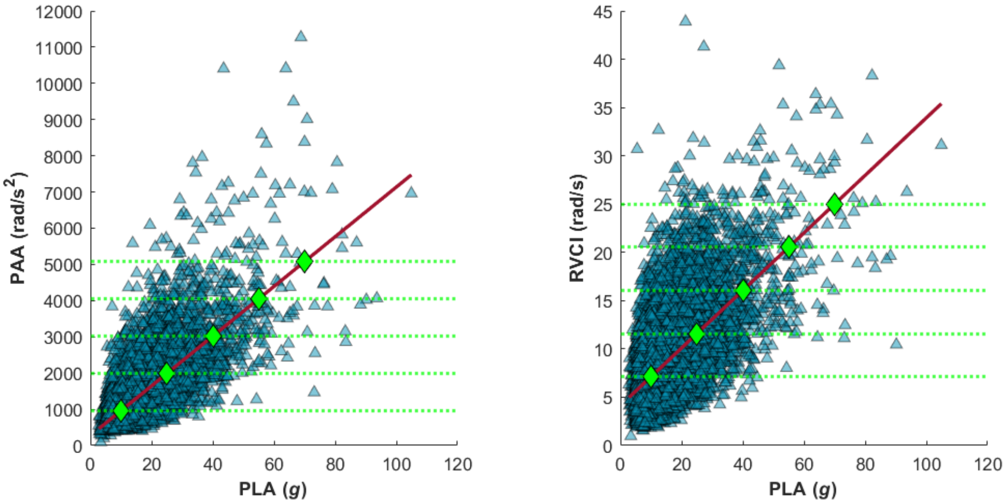

Supplementary Fig. 2 Linear regression between PLA and PAA (a) and RVCI (b). These data were used to determine the approximately proportional thresholds for PAA and RVCI

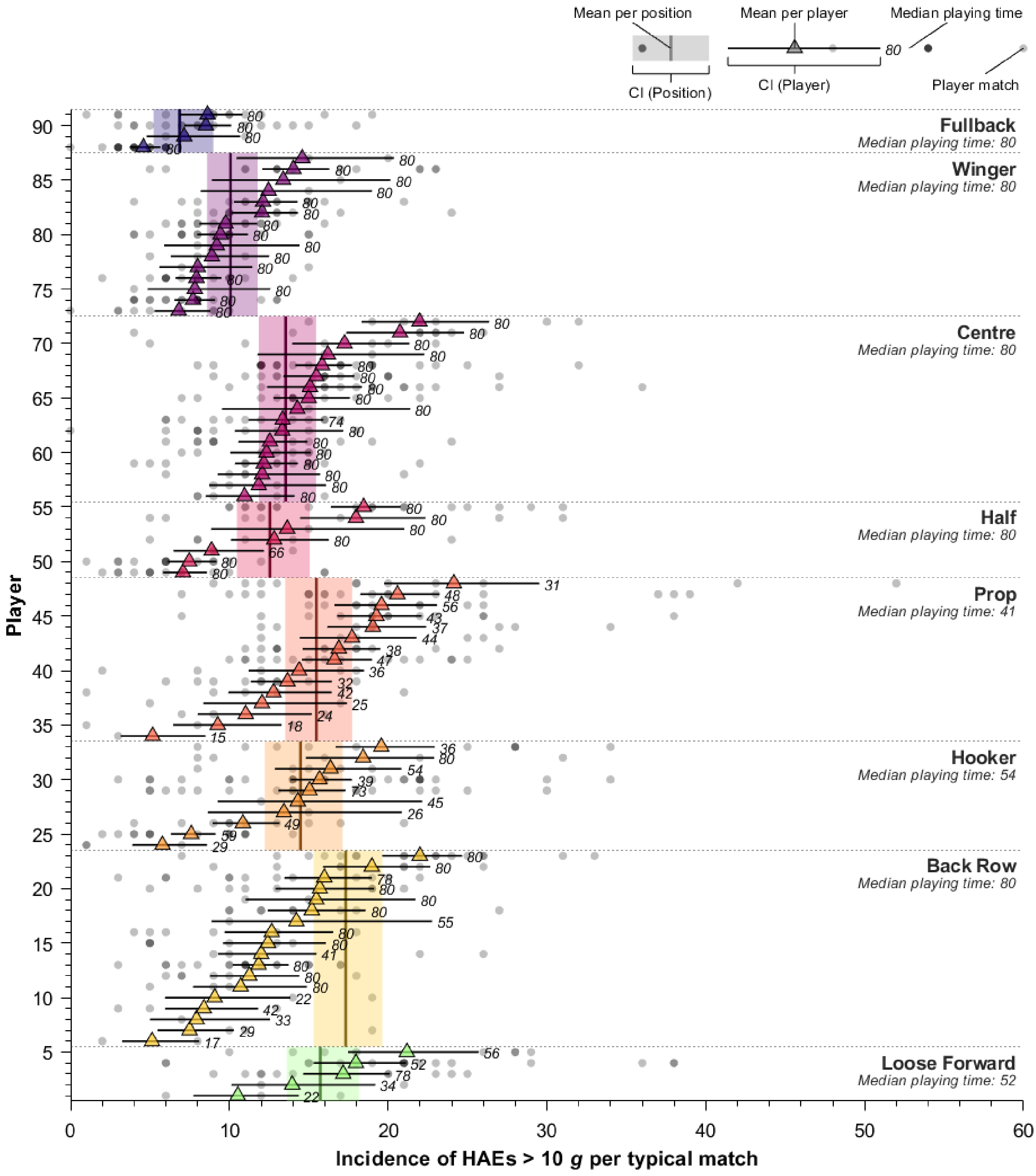

Supplementary Fig. 3 Mean incidence of HAEs exceeding 10 *g per typical match* for each player and each position with individual counts of HAEs per player match.

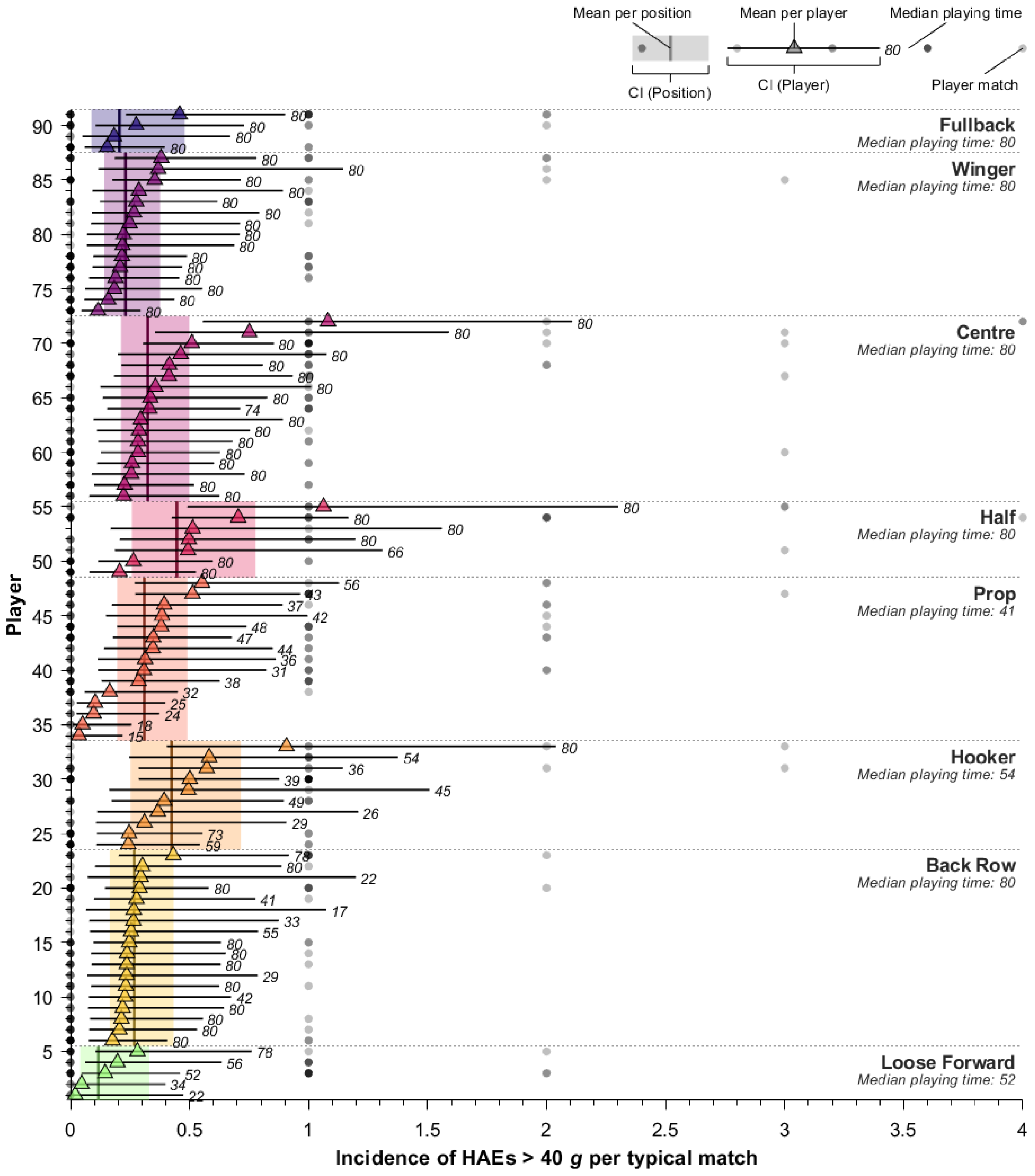

Supplementary Fig. 4 Mean incidence of HAEs exceeding 40 *g per typical match* for each player and each position with individual counts of HAEs per player match.

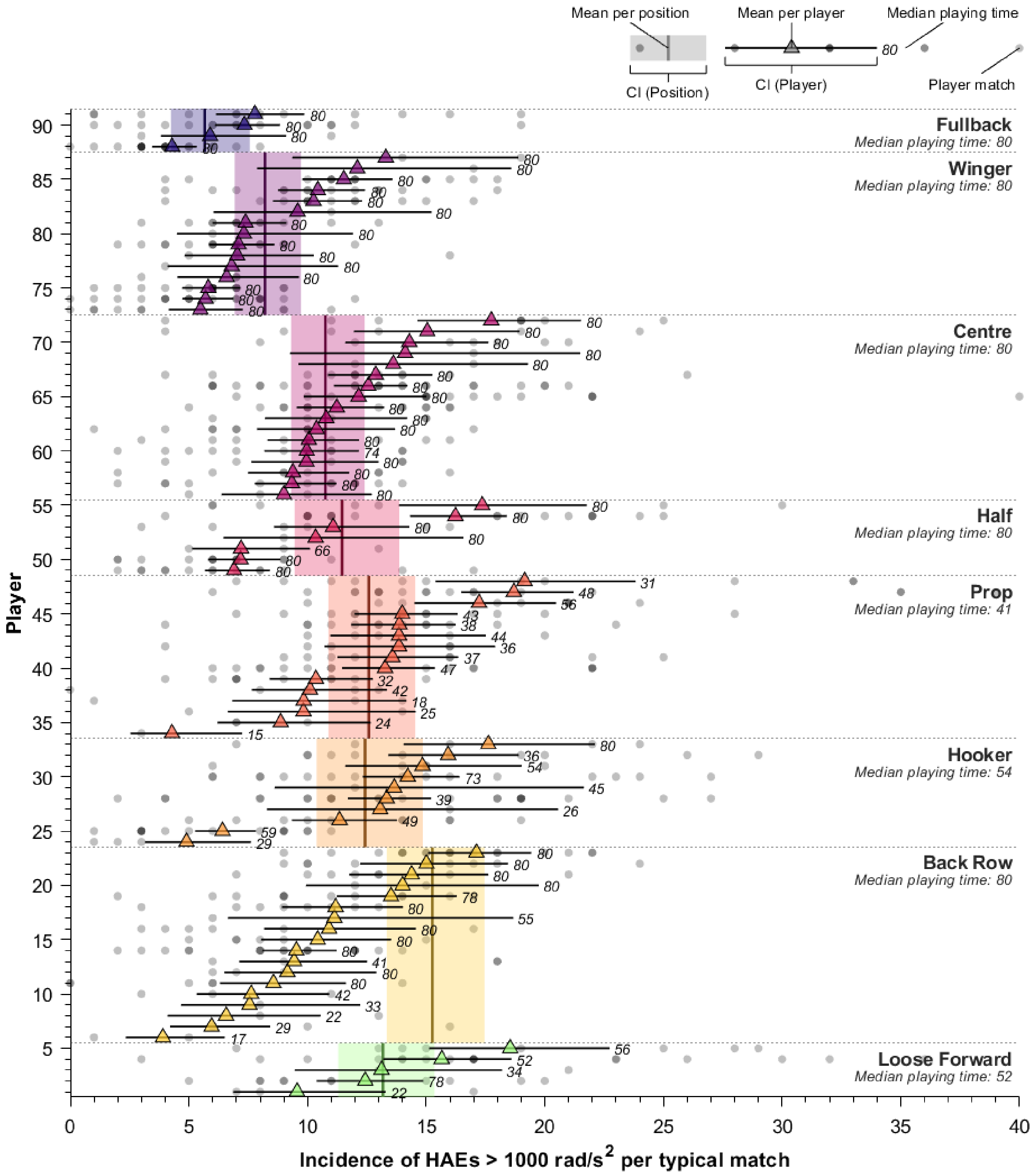

Supplementary Fig. 5 Mean incidence of HAEs exceeding 1,000 rad/s^2 *per typical match* for each player and each position with individual counts of HAEs per player match.

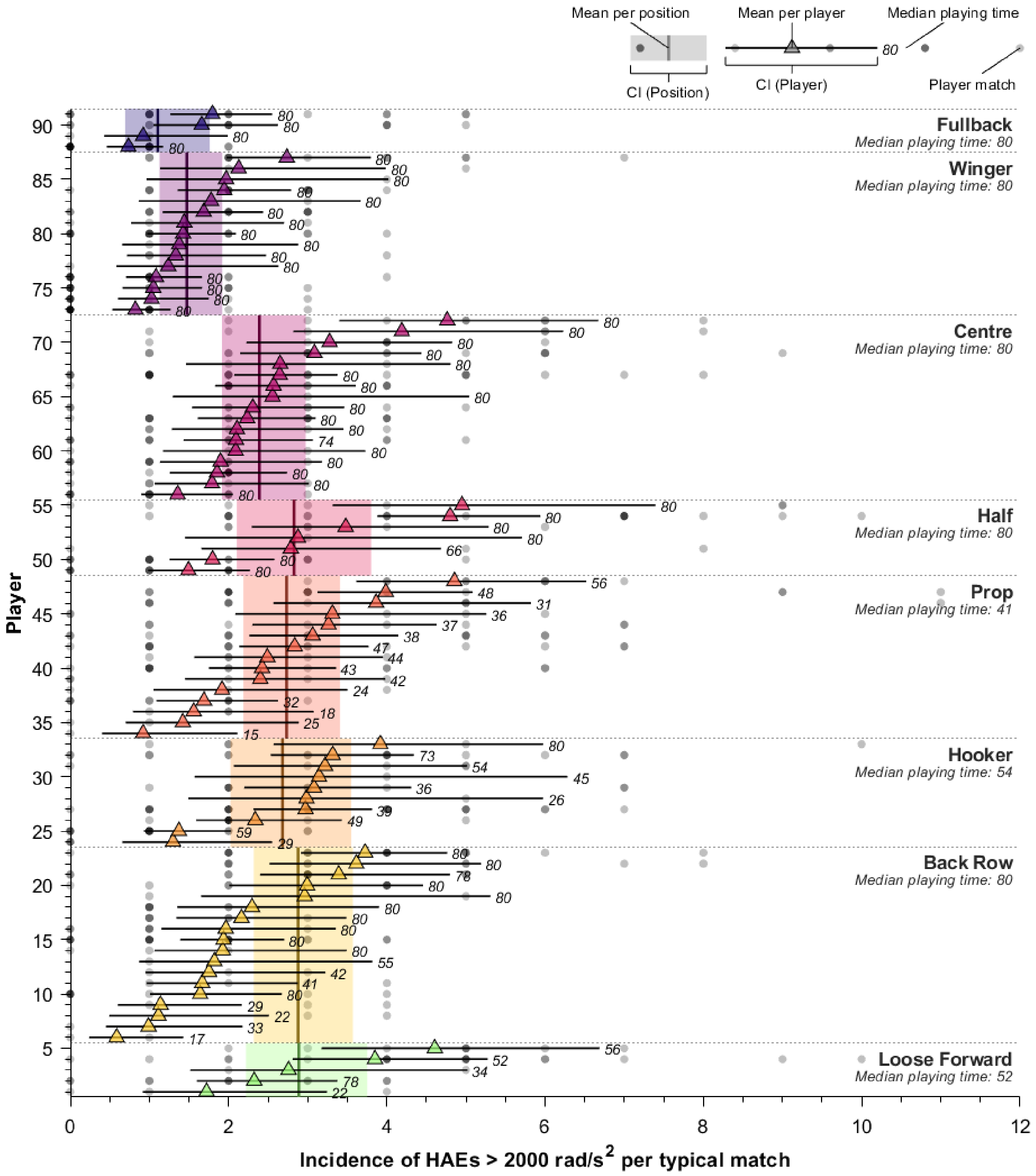

Supplementary Fig. 6 Mean incidence of HAEs exceeding 2,000 rad/s² *per typical match* for each player and each position with individual counts of HAEs per player match.

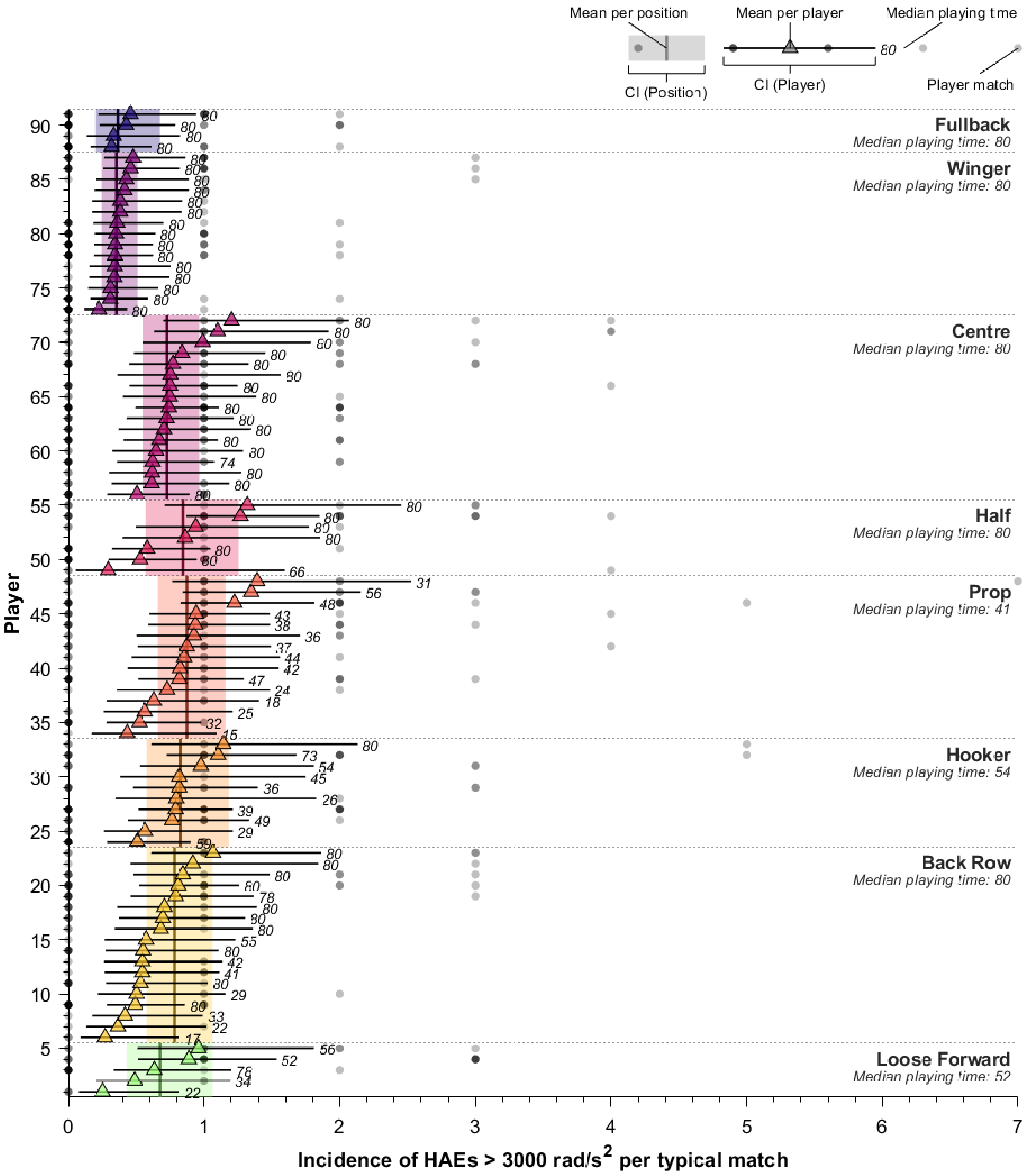

Supplementary Fig. 7 Mean incidence of HAEs exceeding 3,000 rad/s^2 *per typical match* for each player and each position with individual counts of HAEs per player match.

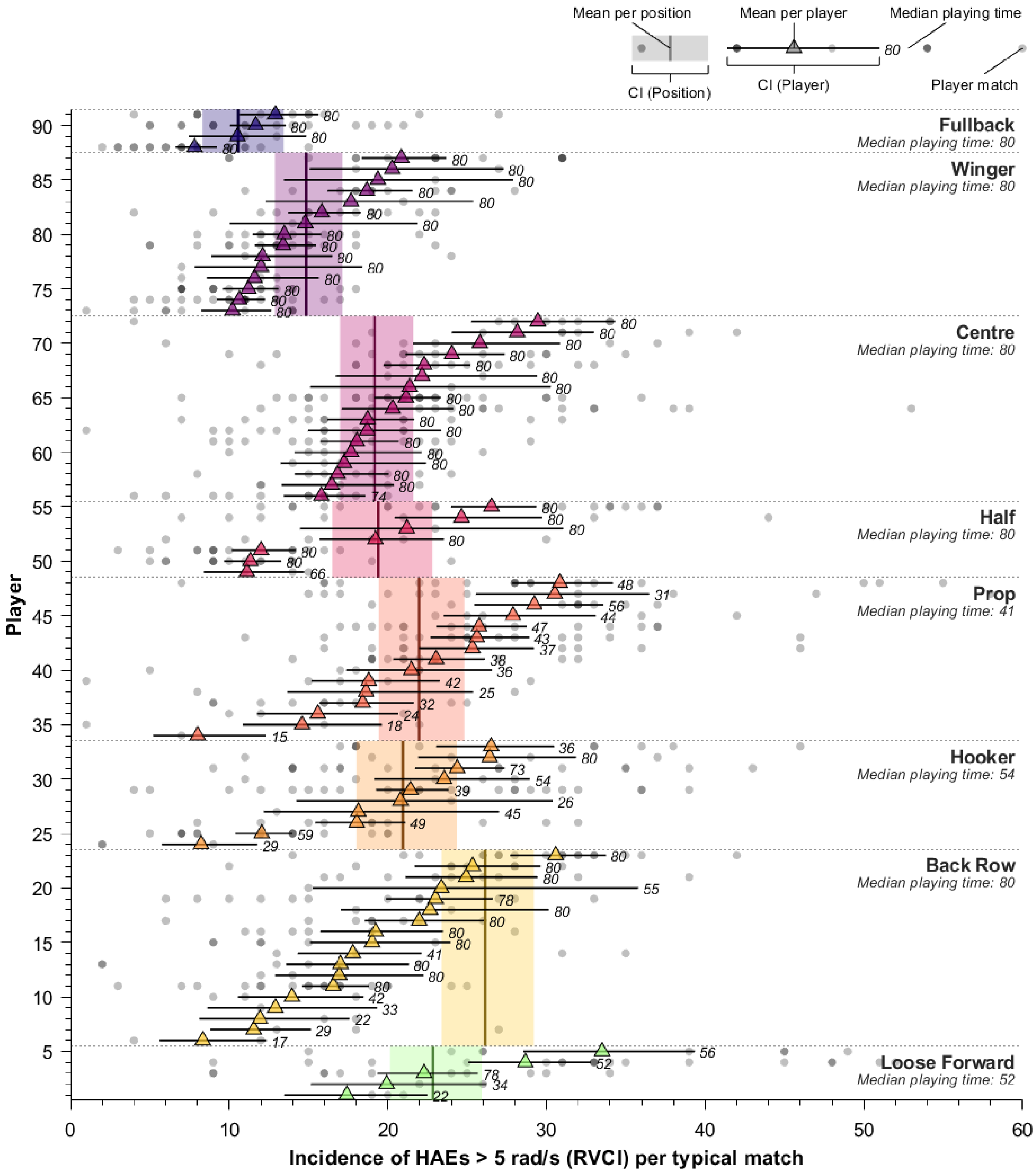

Supplementary Fig. 8 Mean incidence of HAEs exceeding 5 rad/s^2 (RVCI) *per typical match* for each player and each position with individual counts of HAEs per player match.

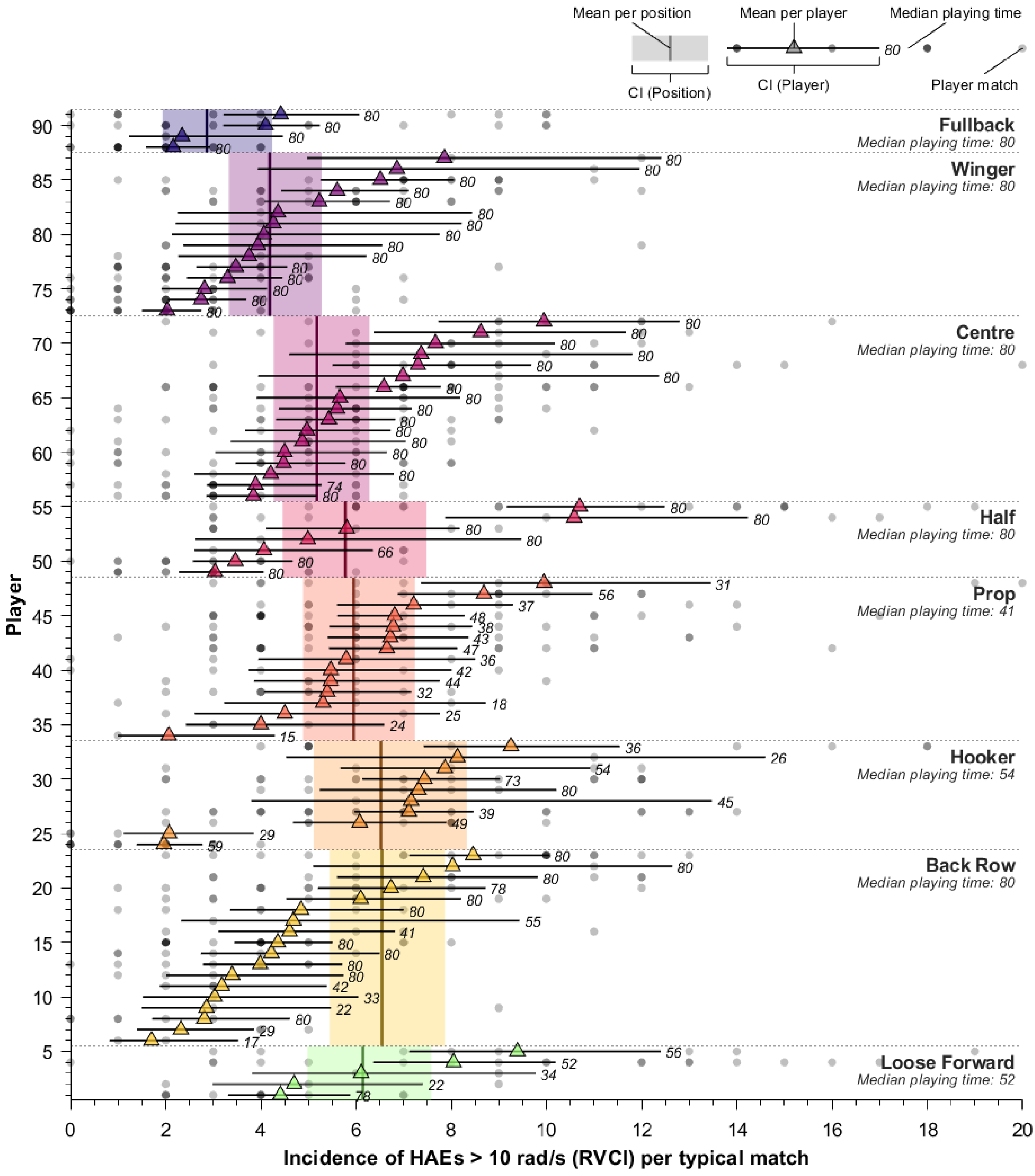

Supplementary Fig. 9 Mean incidence of HAEs exceeding 10 rad/s^2 (RVCI) *per typical match* for each player and each position with individual counts of HAEs per player match.

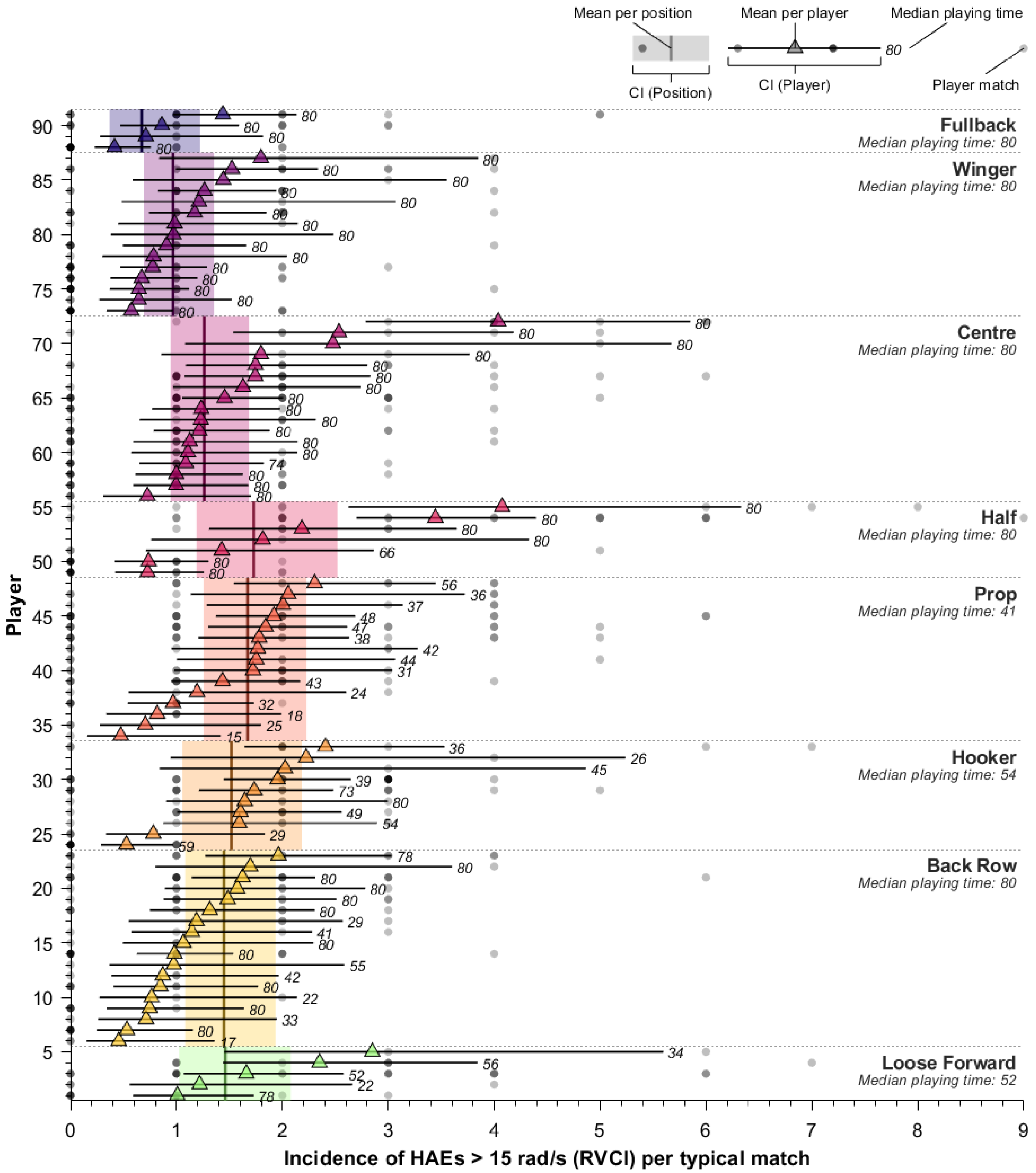

Supplementary Fig. 10 Mean incidence of HAEs exceeding 15 rad/s^2 (RVCI) *per typical match* for each player and each position with individual counts of HAEs per player match.
